# Derivation of a HEAR Pathway for Emergency Department Chest Pain Patients to Safely Avoid a Second Troponin Test

**DOI:** 10.3390/diagnostics13203217

**Published:** 2023-10-16

**Authors:** Chen Chen, Yao Yu, Dongxu Chen, Canguang Cai, Yannan Zhou, Fengqing Liao, Alima Humarbek, Xuan Li, Zhenju Song, Zhan Sun, Chaoyang Tong, Chenling Yao, Guorong Gu

**Affiliations:** Department of Emergency Medicine, Zhongshan Hospital, Fudan University, Shanghai 200032, China; cchen11@fudan.edu.cn (C.C.); yu.yao3@zs-hospital.sh.cn (Y.Y.); chen.dongxu@zs-hospital.sh.cn (D.C.); cai.canguang@zs-hospital.sh.cn (C.C.); zhou.yannan@zs-hospital.sh.cn (Y.Z.); liao.fengqing@zs-hospital.sh.cn (F.L.); a.lima@zs-hospital.sh.cn (A.H.); li.xuan1@zs-hospital.sh.cn (X.L.); song.zhenju@zs-hospital.sh.cn (Z.S.); sun.zhan@zs-hospital.sh.cn (Z.S.); tong.chaoyang@zs-hospital.sh.cn (C.T.)

**Keywords:** HEAR pathway, high-sensitivity cardiac troponin, non-ST elevation myocardial infarction, emergency department, chest pain

## Abstract

The study aims to develop a decision pathway based on HEAR score and 0 h high-sensitivity cardiac troponin T (hs-cTnT) to safely avoid a second troponin test for suspected non-ST elevation myocardial infarction (NSTEMI) in emergency departments. A HEAR score consists of history, electrocardiogram, age, and risk factors. A HEAR pathway is established using a Bayesian approach based on a predefined safety threshold of NSTEMI prevalence in the rule-out group. In total, 7131 patients were retrospectively enrolled, 582 (8.2%) with index visit NSTEMI and 940 (13.2%) with 180-day major adverse cardiovascular events (MACE). For patients with a low-risk HEAR score (0 to 2) and low 0 h hs-cTnT (<14 ng/L), the HEAR pathway recommends early discharge without further testing. After the HEAR pathway had been applied to rule out NSTEMI, the negative predictive value of index visit NSTEMI was 100.0% (95% CI, 99.8% to 100.0%) and false-negative rate of 180-day MACE was 0.40% (95% CI, 0.18% to 0.87%). Compared with the 0 h hs-cTnT < limit of detection (LoD) strategy (<5 ng/L), the HEAR pathway could correctly reclassify 1298 patients without MACE as low risk and lead to a 18.2% decrease (95% CI, 17.4–19.1%) in the need for a second troponin test. The HEAR pathway may lead to a substantial and safe reduction in repeated troponin test for emergency department patients with suspected NSTEMI.

## 1. Introduction

Chest pain is one of the most common reasons for emergency department (ED) visits [1,2]. However, of all ED patients presenting with chest pain, only a small proportion will be diagnosed as having acute coronary syndrome (ACS), and more than half will ultimately be found to have a non-cardiac cause. [2,3]. Rapid triage of ED patients with suspected non-ST elevation myocardial infarction (NSTEMI) remains challenging. On one hand, keeping low-risk patients for repeated tests and prolonged observation can lead to over-testing and ED crowding; on the other hand, delayed diagnosis and treatment of life-threatening conditions can result in significantly elevated short- and long-term mortality [4].

Latest guidelines recommend the use of 0 h/1 h high-sensitivity cardiac troponin (0 h/1 h hs-cTn) algorithm for rapid triage of chest pain patients presenting without persistent ST-segment elevation [5], the safety and efficacy of which have been well-validated in several large multicenter diagnostic studies [5,6]. According to the algorithm, only patients with chest pain onset > 3 h and an initial 0 h hs-cTn below the level of detection (LoD) (e.g., 0 h hs-cTnT < 5 ng/L) can be safely discharged without a second troponin test. Despite guideline recommendations, the ultimate decision on whether and when to order repeated troponin is left to the physician in real-world situations. Notably, in a large U.S. cohort of 27,918 patients, almost half (48.2%) of physicians discharged chest pain patients from the ED after a single troponin test [7]. The study also revealed that the single troponin group was actually a group of patients with lower risk of cardiovascular disease, which indicates that physician’s discretion still plays an important role in clinical decision-making.

However, the use of unstructured risk assessment can result in both under- and over-testing. To improve on this, the 2021 AHA guidelines recommend the use of structured risk assessment tools and evidence-based protocols in clinical practice, incorporating cardiac troponin testing [6]. Several clinical decision pathways based on different structured risk assessment tools have, thus, been developed to help rapid triage of chest pain patients, including the history, electrocardiogram, age, risk factors, and troponin (HEART) score and HEART pathway, the emergency department assessment of chest pain score (EDACS) and EDACS-accelerated diagnostic protocol (EDACS-ADP) [8,9]. The combination of 0 h/1 h hs-cTnT algorithm with HEART pathway or EDACS-ADP enabled the safe ruling out of both index visit AMI and 30-day major adverse cardiac events (MACE) [3,10]. However, unless the patient’s initial troponin level is extremely low (below the LoD), those strategies still require serial troponins to guide disposition, which may lead to downstream unnecessary resource use in low-risk populations with atypical symptoms or those lacking ischemic ECG findings [11,12]. In order to adequately assess the implications of increased testing, we should revisit Bayes’ theorem and its importance in interpreting test results. The Bayesian approach is a quantitative method using a combination of pretest probabilities and the likelihood ratios of diagnostic tests to sequentially refine the post-test probability of a particular disease [13]. In recent years, the Bayesian approach has been used in various clinical scenarios, including interpreting results of COVID-19 screening tests [14,15] and reducing over-testing for suspected pulmonary embolisms [16,17]. Hence, we hypothesized that a clinical decision pathway for ED patients with suspected NSTEMI based on the Bayesian approach would ensure better interpretations of hs-cTnT results and subsequent actions by clinicians.

The history, electrocardiogram, age, and risk factors (HEAR) score (without troponin), deduced from the HEART score, is a risk stratification tool already implemented in clinical practice, and a HEAR score below 2 successfully identifies patients with a very low risk of 30-day MACE [18,19]. The aim of the study was to develop and validate a clinical decision pathway, using the HEAR score as the pretest probability score and 0 h hs-cTnT as the diagnostic test, to safely decrease serial troponin measurements in ED patients with suspected NSTEMI.

## 2. Materials and Methods

### 2.1. Study Subjects

**Figure 1:**
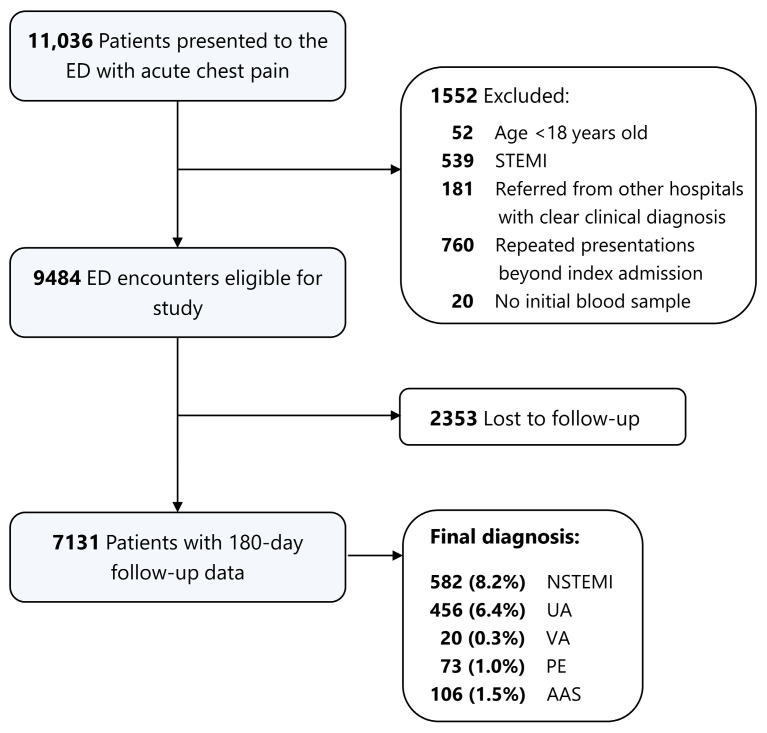
Summary of enrollment, exclusions, and diagnosis, and follow-up. ED, emergency department; STEMI, ST-elevation myocardial infarction; NSTEMI, non-ST-elevation myocardial infarction; UA, unstable angina; VA, variant angina; PE, pulmonary embolism; AAS, acute aortic syndrome.

From 1 January 2019 to 31 December 2019, a total of 11,036 patients presenting with acute chest pain to the emergency department chest pain center of Zhongshan Hospital, Shanghai, China, a tertiary teaching hospital, were screened for this single-center retrospective study. Patients less than 18 years old, diagnosed with ST-elevation myocardial infarction, or referred from other hospitals with clear clinical diagnosis, as well as those repeated presentations beyond index admission, were excluded. Twenty patients without HEART score due to lack of blood samples were also excluded. Finally, 7131 patients with complete 180-day follow-up data were enrolled in the study (Figure 1). The clinical characteristics between patients who were lost to follow-up and those who were successfully followed up are summarized in Table S1. Most of the characteristics show good balance between two groups with a standardized mean difference (SMD) < 0.1. However, patients lost to follow-up were more likely to have a lower HEAR score (4 [IQR: 2–5] vs. 4 [IQR: 3–5], SMD = 0.180, *p* < 0.001) and HEART score (4 [IQR: 2–5] vs. 4 [IQR: 3–6], SMD = 0.202, *p* < 0.001), as well as lower score of item ‘troponin’ (SMD = 0.174, *p* < 0.001). Index visit NSTEMI was lower in patients lost to follow-up (3.2% vs. 8.2%, SMD = 0.216, *p* < 0.001), probably suggesting those lost to follow-up had a lower risk of acute coronary syndrome.

The study was approved by the ethics committee of Zhongshan Hospital Fudan University (Shanghai, China) (approval number B2021-064). Informed consent was obtained from all individuals included in this study.

### 2.2. Laboratory Measurements

Hs-cTnT was measured using the Elecsys^®^ Troponin T high-sensitivity assay (Roche Diagnostics, Basel, Switzerland) with a 99th percentile upper reference limit (URL) of 14 ng/L and a LoD of 5 ng/L. Uniform rather than sex-specific cut-off concentrations of hs-cTnT were used to support the diagnosis of NSTEMI according to current guidelines [5].

Estimated glomerular filtration rate, (eGFR) was calculated based on the Chronic Kidney Disease Epidemiology Collaboration (CKD-EPI) 2021 equation and serum creatinine levels were missing in 2356 patients.

### 2.3. Clinical Data Collection

For index visit, patient demographics, current and past medical history, vital signs, physician examination, and electrocardiogram (ECG) results were reliably contained in the structured electronic medical records and retrospectively collected, reviewed, and analyzed. Follow-up was conducted by telephone interviews using a standardized questionnaire to clarify cardiac events happening since ED discharge and medical practices occurring at other care facilities. Electronic medical record review was used as a surrogate to telephone follow-up for participants unable to be contacted. Participants with no follow-up data available through the phone or from the medical record were considered lost to follow-up.

### 2.4. Risk Assessment Tools

**Figure 2:**
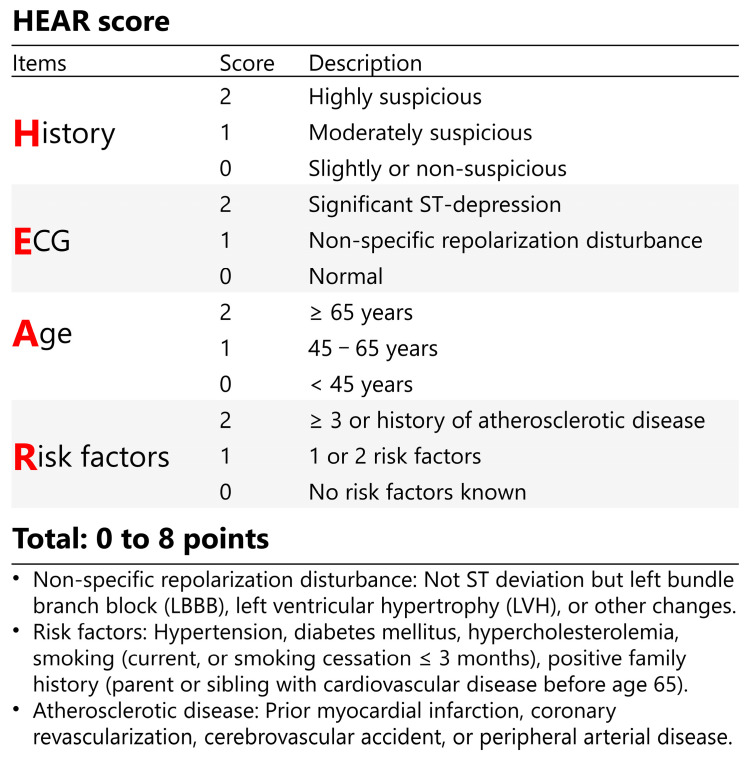
Overview of the HEAR score.

The elements and weights of HEAR score can be found in Figure 2. Calculations of HEART and EDACS scores and procedures of 0 h/1 h hs-cTnT algorithm, HEART pathway, and EDACS-ADP have been previously described [20,21] and are also given in Table 1 and Supplemental Materials.

HEART consists of five elements: history, ECG, age, risk factors, and troponin; each item is given a score of 0 to 2, yielding a total score of 0 to 10. For patients with low-risk HEART scores (0 to 3) and negative serial troponin results (0 h hs-cTnT < 5 ng/or 0 h hs-cTnT < 12 ng/L with a Δ1 h increase <3 ng/L), the HEART pathway recommends direct discharge from ED without further diagnostic testing. HEAR score is deduced from the HEART score, which contains only history, ECG, age, and risk factors.

EDACS consists of age, sex, risk factors, and chest pain characteristics (including pain accompanied with diaphoresis, pain radiating to arm, shoulder, neck or jaw, pain occurred or worsened with inspiration, and pain reproduced by palpation). EDACS-ADP identifies patients as low risk and safe for early discharge if their EDACS < 16 with a non-ischemic ECG and negative serial troponin results (0 h hs-cTnT < 5 ng/L or 0 h hs-cTnT < 12 ng/L with a Δ1 h increase <3 ng/L).

### 2.5. Outcome Measures

The primary outcome measure of interest was the clinical diagnosis of the index visit. NSTEMI was diagnosed based on the current guidelines, which require clinical signs or symptoms of acute myocardial ischemia in combination with evidence of myocardial necrosis, namely, the detection of an increase or decrease in hs-cTnT, with at least one value above the 99th percentile of the upper reference limit, but no persistent ST-segment elevation. Adjudication of the final diagnosis was performed by two independent physicians on the basis of all available medical records obtained during clinical care, including history, physical examination, serial hs-cTnT and other laboratory results, ECG, echocardiography, coronary computed tomography angiography (CCTA), and coronary angiogram findings pertaining to the patient from the time of ED visit to 180-day follow-up. A third cardiologist would be involved in the review and adjudication in case of disagreement on diagnosis.

The secondary outcome measure was MACE within 180 days, which was defined as a composite of cardiac death, non-fatal myocardial infarction (either being the cause for the initial presentation or occurring during the follow-up), and ischemia-driven target lesion revascularization (TLR) (either urgent or elective percutaneous coronary intervention [PCI] or coronary artery bypass grafting [CABG]).

### 2.6. HEAR Pathway Derivation

The Bayesian approach is a quantitative method using a combination of pretest probabilities and the likelihood ratios of diagnostic tests to sequentially refine the post-test probability of a particular disease using deductive reasoning [17]. To derive the HEAR pathway, we predefined the upper limit for NSTEMI prevalence in the rule-out group based on the diagnostic performance of current triage strategies reported in the literature and the prevalence of NSTEMI in the current cohort. In two prior cohorts with moderate NSTEMI prevalence (8% in the first and 7% in the second), the negative predictive ratios (NPV) of 0/1 h and 0/2 h hs-cTn algorithm were 0.998 (0.995–0.999) and 0.996 (0.991–0.999), respectively. Thus, as NSTEMI prevalence in the current study cohort was 8.2%, the acceptable upper limit of NSTEMI prevalence was set at 0.1% for rule-out group to consider the new pathway safe [22,23]. The negative likelihood ratio (NLR) of 0-h hs-cTnT < 14 ng/L was calculated to be 0.025 in the study cohort. Accordingly, to achieve a post-test probability of less than 0.1% with the NLR we calculated, the upper limit of pretest probability can be estimated and the cutoff value of the HEAR score can be achieved. The Fagan’s nomogram was used to provide a visual gauge of how the NLR changes the post-test probability given a particular pretest probability.

Finally, the efficacy of the HEAR pathway was assessed by the rate of second troponin test that could have been avoided if the HEAR pathway had been applied compared with the standard LoD strategy, HEAR pathway, and EDACS-ADP.

### 2.7. Statistical Analysis

Statistical analysis was performed and graphs were constructed using R statistical package (version 4.3.0). Continuous variables were summarized as median and interquartile range (IQR); categorical variables were summarized as number and percentage. Mann–Whitney U test was used to compare continuous variables between groups; chi-square test was used to compare the distribution of categorical variables. Bootstrap resampling with 500 repetitions was used to calculate 95% bias-corrected confidence intervals for the post-test probabilities. Diagnostic sensitivity, specificity, negative predictive value (NPV) and positive predictive value (PPV), and their exact 95% confidence intervals were calculated using the ‘epiR’ package. Different rule-out strategies were compared using McNemar test and net reclassification improvement (NRI). For all analysis, two-tailed probability values were calculated and a *p* value less than 0.05 was considered statistically significant.

## 3. Results

### 3.1. Patient Characteristics

**Figure 3:**
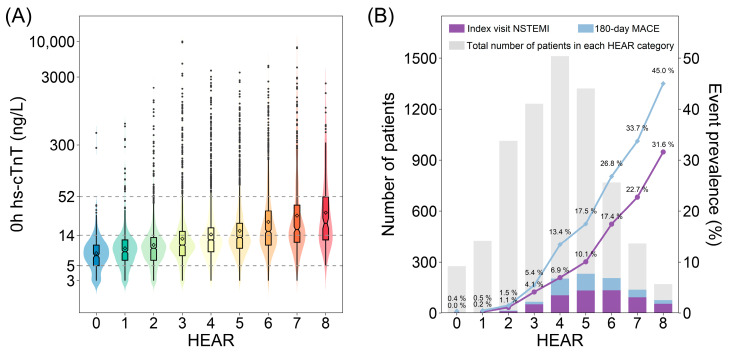
Clinical characteristics of acute chest pain patients with different HEAR scores. (**A**) Distribution of 0 h hs-cTnT and (**B**) prevalence of index visit NSTEMI and 180-day MACE in each HEAR category. In Figure 3A, grey dashed lines were added for reference at specific values of hs-cTnT, i.e., limit of detection (5 ng/L), 99th percentile upper reference limit (14 ng/L), and direct rule-in cut-off (52 ng/L). NSTEMI, non-ST-elevation myocardial infarction; MACE, major adverse cardiac event.

Among 7131 enrolled patients, 582 (8.2%) were diagnosed with NSTEMI at the index visit and 940 (13.2%) had MACE within 180 days. Patient demographic and clinical features are summarized in Table 2. Medium age of the cohort was 64 (IQR: 55–72) years and 3912 patients were male (54.9%). The medium symptom onset time was 2.0 (IQR: 0.1–12.0) h. Patients with a history of coronary artery disease (CAD), AMI, PCI, or CABG accounted for 24.7%, 5.4%, 14.0%, and 0.5%, respectively. As for clinical risk assessment, NSTEMI patients showed remarkably higher HEAR, HEART, and EDACS scores with a median HEAR of 5 (IQR: 4–7), a median HEART of 7 (IQR: 6–8), and a median EDACS of 18 (IQR: 14–21). Initial 0 h hs-cTnT level was significantly higher in NSTEMI patients (119 [47–354] vs. 11 [7–16], *p* < 0.001) and rose in accordance with HEAR scores (r = 0.345, *p* < 0.001) (Figure 3A). HEAR within the study population showed a normal distribution and as HEAR increased, the prevalence of index visit NSTEMI and 180-day MACE accordingly increased (Figure 3B).

### 3.2. Derivation of the HEAR Pathway

**Figure 4:**
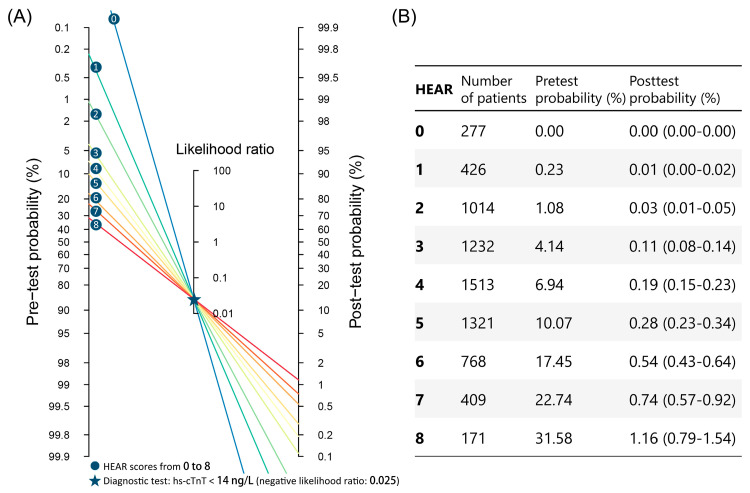
Fagan’s nomogram for patients with suspected NSTEMI. To use the nomogram, draw a straight line from the pre-test probability for the patient (**left-hand side**) through the likelihood ratio for the patient’s test result (**middle**) to arrive at the estimated post-test probability of disease (**right-hand side**). To derive the pathway, pretest probability was calculated based on HEAR score and 0 h hs-cTnT was used as the diagnostic test. Since the negative likelihood ratio (NLR) of 0 h hs-cTnT < 14 ng/L was calculated to be 0.025 in the current cohort, (**A**) Fagan’s nomogram with a NLR of 0.025 and (**B**) the corresponding probability conversion are shown in the figure.

As depicted in the Fagan’s nomogram (Figure 4), given a low enough clinical pretest probability (low HEAR scores) and a negative 0 h hs-cTnT test result, post-test probabilities of NSTEMI could be further reduced. As the acceptable upper limit of the 95% CI of the false-negative rates for rule-out were predefined at 0.1% (see Section 2.6 HEAR Pathway Derivation), a HEAR score of 0 to 2 corresponded to a very low clinical pretest probability. Given a 0 h hs-cTnT < 14 ng/L, the post-test probability of NSTEMI could be decreased to 0.03% (95% CI, 0.01–0.05%).

**Figure 5:**
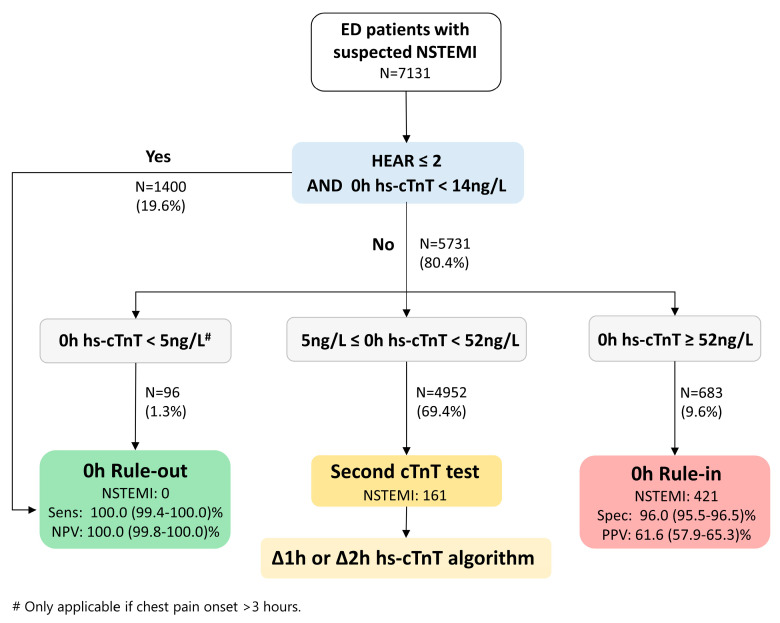
HEAR pathway algorithm and its triage performance. Sens, sensitivity; Spec, specificity; NPV, negative predictive value; PPV, positive predictive value.

On this basis, a new decision pathway, the HEAR pathway, was proposed for rapid triage of ED patients with suspected NSTEMI, aiming to reduce second troponin tests (Figure 5). For patients with low-risk HEAR scores (HEAR 0 to 2) and 0 h hs-cTnT below the 99th percentile URL threshold (0 h hs-cTnT < 14 ng/L), the HEAR pathway recommends rule-out from NSTEMI without further testing. In the rest of the patients, the HEAR pathway recommends triage according to the current 0 h/1 h or 0 h/2 h hs-cTnT algorithm—for those with a very low 0 h hs-cTnT (<5 ng/L), early discharge is appropriate; for those with a significantly elevated 0 h hs-cTnT (>52 ng/L), early cardiology consultation and admission to the hospital are required; for the rest of the patients, prolonged observation and a second troponin test in one or two hours are still needed.

### 3.3. Safety and Efficacy of the HEAR Pathway for Rapid Rule-Out of NSTEMI

The safety and efficacy of the HEAR pathway and other triage strategies for rapid rule-out of NSTEMI are summarized in Table 3. Using the HEAR pathway, 1496 (21.0%) patients would qualify for rule-out after initial clinical assessment and 0 h hs-cTnT measurement; no case of index NSTEMI would be missed, reaching a sensitivity of 100.0% (95% CI, 99.4–100.0%) and an NPV of 100.0% (95% CI, 99.8–100.0%). Six patients (0.4%) with 180-day MACE would be ruled out. The LoD strategy (chest pain onset > 3 h and 0 h hs-cTnT < 5 ng/L) ruled out 196 (2.7%) patients with a sensitivity of 100.0% (95% CI, 99.4–100.0%) and an NPV of 100.0% (95% CI, 98.1–100.0%) for index visit NSTEMI, missing 4 (2.0%) MACE. The HEART pathway ruled-out 315 patients (4.4%) with a sensitivity of 100.0% (95% CI, 99.4–100.0%) and an NPV of 100.0% (95% CI, 98.8–100.0%) for index visit NSTEMI, missing no index visit NSTEMI and 1 (0.3%) MACE. The EDACS-ADP strategy identified 350 (4.9%) patients as low risk with a sensitivity of 100.0% (95% CI, 99.4–100.0%) and an NPV of 100.0% (95% CI, 99.0–100.0%) for index visit NSTEMI, missing no NSTEMI, yet 10 (2.9%) MACE.

The four strategies had comparable safety profiles in terms of ruling out index visit NSTEMI at 0 h; however, the HEAR pathway performed much better in triage efficiency. Compared with the LoD strategy, which is the 0 h rule-out criteria of the 0 h/1 h hs-cTnT algorithm, the HEAR pathway was able to correctly reclassify 1298 patients without 180-day MACE as low risk, yielding a NRI of 20.8% (95% CI, 19.7–21.8%) and leading to a total of 18.2% decrease (95% CI, 17.4–19.1%) in second troponin tests. Compared with the HEART pathway, the HEAR pathway was able to correctly reclassify 1239 patients without 180-day MACE as low risk, yielding a NRI of 18.5% (95% CI, 17.3–19.6%) and leading to a total of 16.6% decrease (95% CI, 15.7–17.4%) in second troponin tests.

### 3.4. Safety and Efficacy of Different Sub-Strategies within the HEAR Pathway

The safety and efficacy of different sub-strategies in the HEAR pathway are summarized in Table 4. It is noteworthy that the sub-strategy ‘HEAR ≤ 2 and 5 ≤ 0 h hs-cTnT < 14 ng/L’ ruled out 1188 (16.7%) patients with no case of index visit NSTEMI missed, reaching a sensitivity of 100.0% (99.4% to 100.0%) and an NPV of 100.0% (99.7% to 100.0%). The results imply that the HEAR pathway is still safe in patients with 0 h hs-cTnT > 5 ng/L, and, more importantly, this sub-strategy yields the highest efficiency among all.

### 3.5. Subgroup Analysis

Subgroup analysis was conducted for chest pain onset (CPO), sex, age, and estimated glomerular filtration rate (eGFR). As shown in Table 5, a significantly larger proportion of patients could be ruled-out at 0 h by the HEAR pathway than by other strategies (i.e., LoD strategy, HEART pathway, and EDACS-ADP) in each and every patient subgroup, including the elderly, those with short symptom onset time, or with renal dysfunction. In addition, more female than male (24.0% vs. 18.7%, *p* = 0.001), more young patients than the elderly (33.5% vs. 5.5%, *p* < 0.001), more patients with longer time from CPO than those with shorter onset (24.7% vs. 18.2%, *p* < 0.001), and more patients with higher eGFR than those with lower eGFR (18.5% vs. 1.7%, *p* < 0.001) were ruled-out by the HEAR pathway.

## 4. Discussion

The initial assessment of ED patients presenting with acute chest pain is focused on the rapid identification of immediately life-threatening conditions, an important one of which is AMI. Using the Bayesian approach and the HEAR risk stratification tool, we derived a new clinical decision pathway to help triage ED patients with suspected NSTEMI. The HEAR pathway performed better than all previously proposed strategies in terms of reducing repeated troponin testing and, consequently, patients’ ED stay time.

Bayes’ theorem is the basic principle that allows physicians to convert the results of a test to the probability of having a disease. At the time of initial patient assessment, physicians will have an unstructured gestalt estimate for the pretest probability of NSTEMI, based on patient demographics, current and past medical history, physical examination, and initial ECG (if available). Subsequent laboratory results including serial troponin tests help physicians update prior information, further increasing or reducing disease probability. Hence, for individuals with different clinical pretest probabilities, the same troponin result can lead to different implications (i.e., post-test probabilities).

Several approaches have been made to accurately assess the clinical pretest probability of patients with suspected NSTEMI. The first one is the gestalt rating scale, which is fast and simple to use. However, it has been proved that clinician gestalt alone is not sufficiently accurate or safe to either rule-in or rule-out ACS [24]. Another proposal is the database-derived attribute matching, which assesses the disease probability in a new patient based upon prior experience with many patients who had similar clinical features as the patient under consideration. Despite validated safety and efficacy, this method is software-dependent, which limits its wide use in clinical practice [25]. EDACS-ADP and the HEART pathway are diagnostic strategies aiming to increase early and safe discharge of low-risk patients, based on clinical risk assessment. In both prospective trials and retrospective external validations, EDACS-ADP identified a substantial proportion (41.6–60.8%) of low-risk patients and showed 99–100% sensitivity for classifying patients as low risk of MACE [26,27,28]. However, since the EDACS generates a score from −8 to 34, it may be difficult to memorize and apply for clinicians.

In recent years, several meta-analyses showed that the pooled sensitivity of the HEART score for predicting 30-day MACEs was above 96% [29,30]. With the combination of hs-cTn-based algorithms, the HEART pathway allowed for an even safer triage of acute chest pain patients [8,31]. Nevertheless, the HEART pathway still largely depends on a second troponin measurement [32], and as a result, its value mainly lies in triage safety but not efficacy. In addition, the HEART score was developed based on the proposer’s own experience and weighting of the score had little rationale. Patients with extremely high and extremely low 0 h hs-cTnT could gain an exact same HEART score. Meanwhile, with the introduction of more sensitive troponin assays, it is becoming more difficult for the HEART pathway to identify low-risk patients for early discharge [33]. Therefore, we believe that the HEAR pathway, which separates 0 h hs-cTnT measurement from initial clinical assessment, could have an advantage over the prior HEART pathway.

Overuse of cardiac troponin is an important concern [34]. Some experts believe that clinicians are able to identify patients at high risk based on detailed and structured clinical risk evaluation beyond the results of a second troponin test [7]. To put this into practice, we used the HEAR score, also known as the modified HEART score, as the clinical risk stratification tool, so that the 0 h hs-cTnT cut-off value could be adjusted according to the pretest probability of specific patient populations, which gave birth to the HEAR pathway. With the use of the HEAR pathway, ED physicians can be guided to rule out more patients with a single troponin test, leading to a reduction in unnecessary resource occupation in low-risk patients. Furthermore, in ED patients presenting with acute chest pain, beyond the presence or absence of AMI, four clinical variables can affect hs-cTn concentrations, namely, sex, age, renal function, and time from CPO [5]. Those characteristics, to a large extent, represent the probability of pre-existing cardiac disease and the difficulty in the differential diagnosis of chest pain. Hence, our results indicate that the HEAR pathway mainly helps accelerate the triage of low-risk patients, namely, those young, female patients without renal dysfunction. A reduction in unnecessary troponin measurement in those low-risk patients could optimize the allocation of medical resources in an ED, and thereby further increase safety for the whole patient population.

Owing to high-sensitivity troponin tests and accelerated diagnostic protocols, the cost for the assessment of acute chest pain has been decreased [35]. However, potential economic burden and increasing ED crowding are still tough problems in developing areas or countries. In China, for example, the number of annual ED visits tripled from 51.9 million to 166.5 million over the past decade, with overcrowding and long length of stay in EDs being the critical challenges [36]. In addition, a recent study showed that a significant proportion of the cost burden related to low-risk patients and non-specific chest pain [37]. Situations became worse in the more recent COVID-19 pandemic, as non-specific chest pain is a commonly-reported long COVID symptom [38]. Hence, the current study tried to further illustrate the question of whether discharge after a single troponin test can be safe in specific clinical settings where reducing repeated test rates may have substantial economic and organizational value.

This study is limited by the single-center, retrospective design, which limits the interpretation and generalization of results. The safety and efficacy of the HEAR pathway need to be validated in external validation cohorts and in prospective studies. Another limitation is that patients lost to follow-up could introduce potential selection bias. In addition, electronic medical record review was used as a surrogate to telephone follow-up for participants unable to be contacted. This could lead to incomplete capture of data on important outcomes, and a lack of representation of vulnerable populations that do not access or receive treatment. Finally, the prevalence of NSTEMI in the study cohort is 8.2%, and, thus, the HEAR pathway should be used with caution in a population of patients with a higher NSTEMI prevalence. Nevertheless, our study has several strengths. First, we used a Bayesian evidence-based medicine approach to establish the clinical decision pathway, based on the predefined safety thresholds. Second, the study cohort derived from a large and accredited chest pain center, where accurate data could be extracted from structured electronic medical records. Although our study was retrospectively designed, clinical data of the index visit were collected in real time. Findings of the current study can form the basis on which prospective studies are designed. In fact, a prospective validation study is ongoing in our center.

## 5. Conclusions

In conclusion, using the Bayesian approach, we derived a new clinical decision pathway, the HEAR pathway, to help ED physicians make decisions regarding patients with suspected NSTEMI. Applying the HEAR pathway may lead to a substantial and safe reduction in repeated troponin tests.

## Figures and Tables

**Table 1:** Rule-out strategies for patients with suspected NSTEMI in emergency department.

Strategies	Clinical Criteria	0 h hs-cTnT Cutoff	0 h/1 h hs-cTnT Cutoff
0 h/1 h hs-cTnT algorithm	Chest pain onset > 3 h (for 0 h rule-out)	0 h hs-cTnT < 5 ng/L	0 h hs-cTnT < 12 ng/L AND Δ1 h hs-cTnT increase < 3 ng/L
HEART pathway	HEART ≤ 3		
EDACS-ADP	EDACS < 16 points AND No signs of acute ischemia on ECG		

NSTEMI, non-ST-elevation myocardial infarction; hs-cTnT, high-sensitivity cardiac troponin T; HEART, history, electrocardiogram, age, risk factors, and troponin; EDACS-ADP, emergency department assessment of chest pain score-accelerated diagnostic protocol.

**Table 2:** Patient characteristics.

Characteristics	Total	Not NSTEMI	NSTEMI	*p* Value
	*N* = 7131	*N* = 6549 (91.8%)	*N* = 582 (8.2%)	
General information				
Age, years	64 [55, 72]	64 [55, 71]	68 [60, 75]	<0.001
Sex, male	3912 (54.9)	3459 (52.8)	453 (77.8)	<0.001
Symptoms				
Symptom onset time, hour	2.0 [0.1, 12.0]	2.0 [0.1, 12.0]	3.0 [0.5, 24.0]	<0.001
Diaphoresis	941 (13.2)	808 (12.3)	133 (22.9)	<0.001
Palpitation	904 (12.7)	867 (13.2)	37 (6.4)	<0.001
Dyspnea	569 (8.0)	510 (7.8)	59 (10.1)	0.054
Signs				
Systolic BP, mmHg	145.0 [129.0, 161.0]	145.0 [129.0, 161.0]	145.0 [129.0, 163.0]	0.723
Diastolic BP, mmHg	79.0 [70.0, 89.0]	79.0 [70.0, 88.0]	78.0 [69.0, 90.0]	0.803
Heart rate, bpm	82.0 [73.0, 93.0]	82.0 [73.0, 93.0]	81.0 [72.0, 92.8]	0.232
Ever smoked	468 (6.6)	390 (6.0)	78 (13.4)	<0.001
History of				
CAD	1759 (24.7)	1563 (23.9)	196 (33.7)	<0.001
AMI	386 (5.4)	328 (5.0)	58 (10.0)	<0.001
PCI	998 (14.0)	878 (13.4)	120 (20.6)	<0.001
CABG	33 (0.5)	26 (0.4)	7 (1.2)	0.015
Hypertension	3474 (48.7)	3103 (47.4)	371 (63.7)	<0.001
Diabetes mellitus	1245 (17.5)	1061 (16.2)	184 (31.6)	<0.001
Risk scores				
HEAR	4 [3, 5]	4 [2, 5]	5 [4, 7]	<0.001
HEART	4 [3, 6]	4 [3, 5]	7 [6, 8]	<0.001
EDACS	14 [10, 18]	14 [10, 18]	18 [14, 21]	<0.001
HEART components				
Item: History				<0.001
Score 0	2524 (35.4)	2461 (37.6)	63 (10.8)	
Score 1	1329 (18.6)	1192 (18.2)	137 (23.5)	
Score 2	3278 (46.0)	2896 (44.2)	382 (65.6)	
Item: ECG				<0.001
Score 0	4546 (63.7)	4352 (66.5)	194 (33.3)	
Score 1	830 (11.6)	719 (11.0)	111 (19.1)	
Score 2	1755 (24.6)	1478 (22.6)	277 (47.6)	
Item: Age				<0.001
Score 0	1008 (14.1)	977 (14.9)	31 (5.3)	
Score 1	2645 (37.1)	2452 (37.4)	193 (33.2)	
Score 2	3478 (48.8)	3120 (47.6)	358 (61.5)	
Item: Risk factors				<0.001
Score 0	2744 (38.5)	2638 (40.3)	106 (18.2)	
Score 1	2954 (41.4)	2675 (40.8)	279 (47.9)	
Score 2	1433 (20.1)	1236 (18.9)	197 (33.8)	
Item: Initial troponin				<0.001
Score 0	4431 (62.1)	4421 (67.5)	10 (1.7)	
Score 1	1892 (26.5)	1778 (27.1)	114 (19.6)	
Score 2	808 (11.3)	350 (5.3)	458 (78.7)	
0-h hs-cTnT, ng/L	11 [8, 18]	11 [7, 16]	119 [47, 354]	<0.001

Continuous variables were summarized as median (interquartile range) and categorical variable were summarized as number (percentage). NSTEMI, non-ST-elevation myocardial infarction; BP, blood pressure; CAD, coronary heart disease; AMI, acute myocardial infarction; PCI, percutaneous coronary intervention; CABG, coronary artery bypass graft; EDACS, emergency department assessment of chest pain score; HEAR, history, electrocardiogram, age, and risk factors; HEART, history, electrocardiogram, age, risk factors, and troponin; ECG, electrocardiogram; hs-cTnT, high-sensitivity cardiac troponin T.

**Table 3:** Safety and efficacy of different 0 h rule-out strategies.

0-h Rule-Out Strategies	HEAR Pathway	LoD Strategy ^1^	HEART Pathway	EDACS-ADP
Patients ruled-out, *n* (%)	1496 (21.0)	196 (2.7)	315 (4.4)	350 (4.9)
Index visit NSTEMI, *n* (%)	0 (0.0)	0 (0.0)	0 (0.0)	0 (0.0)
Sensitivity, %	100.0 (99.4–100.0)	100.0 (99.4–100.0)	100.0 (99.4–100.0)	100.0 (99.4–100.0)
NPV, %	100.0 (99.8–100.0)	100.0 (98.1–100.0)	100.0 (98.8–100.0)	100.0 (99.0–100.0)
180-day MACE, *n* (%)	6 (0.4)	4 (2.0)	1 (0.3)	10 (2.9)
Sensitivity, %	99.4 (98.6–99.8)	99.6 (98.9–99.9)	99.8 (99.2–100.0)	99.9 (99.4–100.0)
NPV, %	99.6 (99.1–99.9)	98.0 (94.9–99.4)	99.9 (99.5–100.0)	99.7 (98.2–100.0)
180-day MACE components, *n* (%)				
Cardiac death, *n* (%)	0 (0.0)	0 (0.0)	0 (0.0)	0 (0.0)
Non-fatal STEMI, *n* (%)	1 (0.1)	0 (0.0)	1 (0.3)	1 (0.3)
Non-fatal NSTEMI, *n* (%)	0 (0.0)	0 (0.0)	0 (0.0)	0 (0.0)
Ischemia-driven TLR, *n* (%)	5 (0.3)	4 (2.0)	1 (0.3)	10 (2.9)

^1^ Only applicable if chest pain onset > 3 h. NSTEMI, non-ST-elevation myocardial infarction; NPV, negative predictive value; MACE, major adverse cardiac events; STEMI, ST-elevation myocardial infarction; TLR, target lesion revascularization; HEAR, history, electrocardiogram, age, and risk factors; LoD, limit of detection; HEART, history, electrocardiogram, age, risk factors, and troponin; EDACS-ADP, emergency department assessment of chest pain score-accelerated diagnostic protocol.

**Table 4:** Triage efficacy and safety of different sub-strategies within the HEAR pathway.

0-h Rule-Out Strategies	HEAR Pathway	Sub-Strategies		
		① HEAR ≤ 2 AND 0 h hs-cTnT < 5 ng/L	② HEAR ≤ 2 AND 5 ≤ 0 h hs-cTnT < 14 ng/L	③ HEAR > 2 AND 0 h hs-cTnT < 5 ng/L ^1^
Patients ruled-out, *n* (%)	1496 (21.0)	212 (3.0)	1188 (16.7)	96 (1.3)
Index visit NSTEMI, *n* (%)	0 (0.0)	0 (0.0)	0 (0.0)	0 (0.0)
Sensitivity, %	100.0 (99.4–100.0)	100.0 (99.4, 100.0)	100.0 (99.4, 100.0)	100.0 (99.4, 100.0)
NPV, %	100.0 (99.8–100.0)	100.0 (98.3, 100.0)	100.0 (99.7, 100.0)	100.0 (96.2, 100.0)
180-day MACE, *n* (%)	6 (0.4)	0 (0.0)	2 (0.2)	4 (4.2)
Sensitivity, %	99.4 (98.6–99.8)	100.0 (99.6, 100.0)	99.8 (99.2, 100.0)	99.6 (98.9–99.9)
NPV, %	99.6 (99.1–99.9)	100.0 (98.3, 100.0)	99.8 (99.4, 100.0)	95.8 (89.7–98.9)
180-day MACE events, *n* (%)				
Cardiac death, *n* (%)	0 (0.0)	0 (0.0)	0 (0.0)	0 (0.0)
Non-fatal STEMI, *n* (%)	1 (0.1)	0 (0.0)	1 (0.1)	0 (0.0)
Non-fatal NSTEMI, *n* (%)	0 (0.0)	0 (0.0)	0 (0.0)	0 (0.0)
Ischemia-driven TLR, *n* (%)	5 (0.3)	0 (0.0)	1 (0.1)	4 (4.2)

^1^ Only applicable if chest pain onset > 3 h. NSTEMI, non-ST-elevation myocardial infarction; NPV, negative predictive value; MACE, major adverse cardiac events; STEMI, ST-elevation myocardial infarction; TLR, target lesion revascularization; HEAR, history, electrocardiogram, age, and risk factors; hs-cTnT, high-sensitivity cardiac troponin T.

**Table 5:** Subgroup analysis of the triage efficacy of different 0 h rule-out strategies.

Patient Characteristics, *n*			Patients Ruled-Out, *n* (%)			
			HEAR Pathway	LoD Strategy ^1^	HEART Pathway	EDACS-ADP
Total		*n* = 7131	1496 (21.0)	196 (2.7) ***	315 (4.4) ***	350 (4.9) ***
CPO	≤3 h	*n* = 4077	741 (18.2)	/	176 (4.3) ***	205 (5.0) ***
	>3 h	*n* = 3054	755 (24.7)	196 (6.4) ***	139 (4.6) ***	145 (4.7) ***
Sex	Females	*n* = 3219	773 (24.0)	134 (4.2) ***	203 (6.3) ***	245 (7.6) ***
	Males	*n* = 3912	723 (18.5)	62 (1.6) ***	112 (2.9) ***	105 (2.7) ***
Age	≤65 years	*n* = 3938	1321 (33.5)	166 (4.2) ***	294 (7.5) ***	314 (8.0) ***
	>65 years	*n* = 3193	175 (5.5)	30 (0.9) ***	21 (0.7) ***	36 (1.1) ***
eGFR ^2^	<60 mL·min^−1^·1.73 m^2^	*n* = 528	9 (1.7)	1 (0.2) *	0 (0.0) *	1 (0.2) *
	>60 mL·min^−1^·1.73 m^2^	*n* = 4247	785 (18.5)	97 (2.3) ***	155 (3.6) ***	173 (4.1) ***

^1^ Only applicable if chest pain onset > 3 h. ^2^ Serum creatinine and eGFR were missing in 2356 patients. CPO, chest pain onset; eGFR, estimated glomerular filtration rate; HEAR, history, electrocardiogram, age, and risk factors; LoD, limit of detection; HEART, history, electrocardiogram, age, risk factors, and troponin; EDACS-ADP, emergency department assessment of chest pain score-accelerated diagnostic protocol. * *p* < 0.05, *** *p* < 0.001. *p* values are calculated from McNemar test assessing the difference between proportion of patients ruled-out by HEAR pathway and by other 0 h rule-out strategies (i.e., LoD strategy, HEART pathway, and EDACS-ADP).

## Data Availability

The datasets used and/or analyzed during the current study are available from the corresponding authors on reasonable request.

## Supplementary Materials

The following supporting information can be downloaded at: https://www.mdpi.com/article/10.3390/diagnostics13203217/s1, Table S1: Analysis of clinical characteristics between patients who were lost to follow-up and those who were successfully followed up; Table S2: Calculation of HEART score; Table S3: Calculation of EDACS.

## References

[1] Curfman G. (2018). Acute Chest Pain in the Emergency Department. JAMA Intern. Med..

[2] Hsia R.Y., Hale Z., Tabas J.A. (2016). A National Study of the Prevalence of Life-Threatening Diagnoses in Patients with Chest Pain. JAMA Intern. Med..

[3] Ekelund U., Akbarzadeh M., Khoshnood A., Björk J., Ohlsson M. (2012). Likelihood of acute coronary syndrome in emergency department chest pain patients varies with time of presentation. BMC Res. Notes.

[4] Mehta R.H., Eagle K.A. (2000). Missed Diagnoses of Acute Coronary Syndromes in the Emergency Room—Continuing Challenges. New Engl. J. Med..

[5] Collet J.P., Thiele H., Barbato E., Barthélémy O., Bauersachs J., Bhatt D.L., Dendale P., Dorobantu M., Edvardsen T., Folliguet T. (2021). 2020 ESC Guidelines for the management of acute coronary syndromes in patients presenting without persistent ST-segment elevation. Eur. Heart J..

[6] Gulati M., Levy P.D., Mukherjee D., Amsterdam E., Bhatt D.L., Birtcher K.K., Blankstein R., Boyd J., Bullock-Palmer R.P., Conejo T. (2021). 2021 AHA/ACC/ASE/CHEST/SAEM/SCCT/SCMR Guideline for the Evaluation and Diagnosis of Chest Pain: Executive Summary: A Report of the American College of Cardiology/American Heart Association Joint Committee on Clinical Practice Guidelines. Circulation.

[7] Wassie M., Lee M.-S., Sun B.C., Wu Y.-L., Baecker A.S., Redberg R.F., Ferencik M., Shen E., Musigdilok V., Sharp A.L. (2021). Single vs Serial Measurements of Cardiac Troponin Level in the Evaluation of Patients in the Emergency Department with Suspected Acute Myocardial Infarction. JAMA Netw. Open.

[8] Mahler S.A., Riley R.F., Hiestand B.C., Russell G.B., Hoekstra J.W., Lefebvre C.W., Nicks B.A., Cline D.M., Askew K.L., Elliott S.B. (2015). The HEART Pathway randomized trial: Identifying emergency department patients with acute chest pain for early discharge. Circ. Cardiovasc. Qual. Outcomes.

[9] Than M.P., Pickering J.W., Dryden J.M., Lord S.J., Aitken S.A., Aldous S.J., Allan K.E., Ardagh M.W., Bonning J.W., Callender R. (2018). ICare-ACS (Improving Care Processes for Patients with Suspected Acute Coronary Syndrome): A Study of Cross-System Implementation of a National Clinical Pathway. Circulation.

[10] Nilsson T., Johannesson E., Lundager Forberg J., Mokhtari A., Ekelund U. (2021). Diagnostic accuracy of the HEART Pathway and EDACS-ADP when combined with a 0-h/1-h hs-cTnT protocol for assessment of acute chest pain patients. Emerg. Med. J. EMJ.

[11] Shimoni Z., Arbuzov R., Froom P. (2017). Troponin Testing in Patients without Chest Pain or Electrocardiographic Ischemic Changes. Am. J. Med..

[12] Sandoval Y., Gunsolus I.L., Smith S.W., Sexter A., Thordsen S.E., Carlson M.D., Johnson B.K., Bruen C.A., Dodd K.W., Driver B.E. (2019). Appropriateness of Cardiac Troponin Testing: Insights from the Use of TROPonin In Acute coronary syndromes (UTROPIA) Study. Am. J. Med..

[13] Quintana M., Viele K., Lewis R.J. (2017). Bayesian Analysis: Using Prior Information to Interpret the Results of Clinical Trials. JAMA.

[14] Chan G.M. (2020). Bayes’ theorem, COVID-19, and screening tests. Am. J. Emerg. Med..

[15] Bell K.J., Stanaway F.F., Irwig L.M., Horvath A.R., Teixeira-Pinto A., Loy C. (2021). How to use imperfect tests for COVID-19 (SARS-CoV-2) to make clinical decisions. Med. J. Aust..

[16] Hobensack M., Phan N. (2021). Derivation and Validation of a 4-Level Clinical Pretest Probability Score for Suspected Pulmonary Embolism to Safely Decrease Imaging Testing. J. Emerg. Med..

[17] Carpenter C.R., Raja A.S. (2014). Arming the Bayesian physician to rule out pulmonary embolism: Using evidence-based diagnostics to combat overtesting. Acad. Emerg. Med..

[18] Moumneh T., Sun B.C., Baecker A., Park S., Redberg R., Ferencik M., Lee M.-S., Douillet D., Roy P.-M., Sharp A.L. (2020). Identifying Patients with Low Risk of Acute Coronary Syndrome Without Troponin Testing: Validation of the HEAR Score. Am. J. Med..

[19] Smith L.M., Ashburn N.P., Snavely A.C., Stopyra J.P., Lenoir K.M., Wells B.J., Hiestand B.C., Herrington D.M., Miller C.D., Mahler S.A. (2020). Identification of very low-risk acute chest pain patients without troponin testing. Emerg. Med. J..

[20] Stepinska J., Lettino M., Ahrens I., Bueno H., Garcia-Castrillo L., Khoury A., Lancellotti P., Mueller C., Muenzel T., Oleksiak A. (2020). Diagnosis and risk stratification of chest pain patients in the emergency department: Focus on acute coronary syndromes. A position paper of the Acute Cardiovascular Care Association. Eur. Hear. J. Acute Cardiovasc. Care.

[21] Than M., Flaws D., Sanders S., Doust J., Glasziou P., Kline J., Aldous S., Troughton R., Reid C., A Parsonage W. (2014). Development and validation of the Emergency Department Assessment of Chest pain Score and 2 h accelerated diagnostic protocol. Emerg. Med. Australas..

[22] Reichlin T., Cullen L., Parsonage W.A., Greenslade J., Twerenbold R., Moehring B., Wildi K., Mueller S., Zellweger C., Mosimann T. (2014). Two-hour Algorithm for Triage Toward Rule-out and Rule-in of Acute Myocardial Infarction Using High-sensitivity Cardiac Troponin T. Am. J. Med..

[23] Chapman A.R., Hesse K., Andrews J., Ken Lee K., Anand A., Shah A.S., Sandeman D., Ferry A.V., Jameson J., Piya S. (2018). High-Sensitivity Cardiac Troponin I and Clinical Risk Scores in Patients with Suspected Acute Coronary Syndrome. Circulation.

[24] Kline J.A., Stubblefield W.B. (2014). Clinician Gestalt Estimate of Pretest Probability for Acute Coronary Syndrome and Pulmonary Embolism in Patients with Chest Pain and Dyspnea. Ann. Emerg. Med..

[25] A Kline J., Johnson C.L., PollackJr C.V., Diercks D.B., E Hollander J., Newgard C.D., Garvey J.L. (2005). Pretest probability assessment derived from attribute matching. BMC Med. Inform. Decis. Mak..

[26] Flaws D., Than M., Scheuermeyer F.X., Christenson J., Boychuk B., Greenslade J.H., Aldous S., Hammett C.J., A Parsonage W., Deely J.M. (2016). External validation of the emergency department assessment of chest pain score accelerated diagnostic pathway (EDACS-ADP). Emerg. Med. J..

[27] Mark D.G., Huang J., Chettipally U., Kene M.V., Anderson M.L., Hess E.P., Ballard D.W., Vinson D.R., Reed M.E. (2018). Performance of Coronary Risk Scores Among Patients with Chest Pain in the Emergency Department. J. Am. Coll. Cardiol..

[28] Than M.P., Pickering J.W., Aldous S.J., Cullen L., Frampton C.M., Peacock W.F., Jaffe A.S., Goodacre S.W., Richards A.M., Ardagh M.W. (2016). Effectiveness of EDACS Versus ADAPT Accelerated Diagnostic Pathways for Chest Pain: A Pragmatic Randomized Controlled Trial Embedded Within Practice. Ann. Emerg. Med..

[29] van den Berg P., Body R. (2018). The HEART score for early rule out of acute coronary syndromes in the emergency department: A systematic review and meta-analysis. Eur. Heart J. Acute Cardiovasc. Care.

[30] Ke J., Chen Y., Wang X., Wu Z., Chen F. (2021). Indirect comparison of TIMI, HEART and GRACE for predicting major cardiovascular events in patients admitted to the emergency department with acute chest pain: A systematic review and meta-analysis. BMJ Open.

[31] Mahler S.A., Lenoir K.M., Wells B.J., Burke G.L., Duncan P.W., Case L.D., Herrington D.M., Diaz-Garelli J.F., Futrell W.M., Hiestand B.C. (2018). Safely Identifying Emergency Department Patients with Acute Chest Pain for Early Discharge. Circulation.

[32] Mahler S.A., Stopyra J.P., Apple F.S., Riley R.F., Russell G.B., Hiestand B.C., Hoekstra J.W., Lefebvre C.W., Nicks B.A., Cline D.M. (2017). Use of the HEART Pathway with high sensitivity cardiac troponins: A secondary analysis. Clin. Biochem..

[33] Aung S.S.M., Roongsritong C. (2022). A Closer Look at the HEART Score. Cardiol. Res..

[34] Morgan D.J., Dhruva S.S., Coon E.R., Wright S.M., Korenstein D. (2019). 2018 Update on Medical Overuse. JAMA Intern. Med..

[35] Jülicher P., Greenslade J.H., A Parsonage W., Cullen L. (2017). The organisational value of diagnostic strategies using high-sensitivity troponin for patients with possible acute coronary syndromes: A trial-based cost-effectiveness analysis. BMJ Open.

[36] Pan C., Pang J.-J., Cheng K., Xu F., Chen Y.-G. (2021). Trends and challenges of emergency and acute care in Chinese mainland: 2005–2017. World J. Emerg. Med..

[37] Dawson L., Nehme E., Nehme Z., Zomer E., Bloom J., Cox S., Anderson D., Stephenson M., Lefkovits J., Taylor A. (2023). Healthcare cost burden of acute chest pain presentations. Emerg. Med. J..

[38] Huang L., Li X., Gu X., Zhang H., Ren L., Guo L., Liu M., Wang Y., Cui D., Wang Y. (2022). Health outcomes in people 2 years after surviving hospitalisation with COVID-19: A longitudinal cohort study. Lancet Respir. Med..

