# Supplementary material for: Derivation of a HEAR Pathway for Emergency Department Chest Pain Patients to Safely Avoid a Second Troponin Test
Source: Diagnostics (Basel). 2023 Oct 16;13(20):3217. doi: 10.3390/diagnostics13203217 (PMC10605779; doi:10.3390/diagnostics13203217)
Supplement: Supplementary file 1 [file diagnostics-13-03217-s001.zip › diagnostics-2556965-supplementary.pdf]

Supplementary Materials

Derivation of a HEAR Pathway for Emergency Department Chest Pain Patients to Safely Avoid a Second Troponin Test

Chen Chen, Yao Yu, Dongxu Chen, Canguang Cai, Yannan Zhou, Fengqing Liao, Alima Humarbek, Xuan Li, Zhenju Song, Zhan Sun, Chaoyang Tong, Chenling Yao* and Guorong Gu*

***Correspondence**: Prof. Guorong Gu (gu.guorong@zs-hospital.sh.cn) and Prof. Chenling Yao (yao.chenling@zs-hospital.sh.cn)

Table S1. Analysis of clinical characteristics between patients who were lost to follow-up and those who were successfully followed up.

Characteristics	Overall<br>9484	Lost to follow-up<br>*N*=2353	Successfully followed up<br>*N*=7131	SMD
General information				
Age, years	64.000 [55, 71]	64.000 [55, 71]	64 [55, 72]	0.021
Sex, male	5106 (53.8)	1194 (50.7)	3912 (54.9)	0.083
Symptoms				
Symptom onset time, hr	3.0 [0.3, 12.0]	4.0 [0.5, 24.0]	2.0 [0.1, 12.0]	0.062
Diaphoresis	1195 (12.6)	254 (10.8)	941 (13.2)	0.074
Palpitation	1241 (13.1)	337 (14.3)	904 (12.7)	0.048
Dyspnea	771 (8.1)	202 (8.6)	569 (8.0)	0.022
Signs				
Systolic BP, mmHg	145.0 [129.0, 161.0]	145.000 [129.0, 162.0]	145.0 [129.0, 161.0]	0.014
Diastolic BP, mmHg	79.0 [70.0, 89.0]	80.0 [70.0, 89.0]	79.0 [70.0, 89.0]	0.032
Heart rate, bpm	82.0 [73.0, 93.0]	83.0 [73.0, 94.0]	82.0 [73.0, 93.0]	0.038
Ever smoker	637 (6.7)	169 (7.2)	468 (6.6)	0.024
History of				
CAD	2244 (23.7)	485 (20.6)	1759 (24.7)	0.097
AMI	489 (5.2)	103 (4.4)	386 (5.4)	0.048
PCI	1263 (13.3)	265 (11.3)	998 (14.0)	0.082
CABG	49 (0.5)	16 (0.7)	33 (0.5)	0.029
Hypertension	4557 (48.0)	1083 (46.0)	3474 (48.7)	0.054
Diabetes mellitus	1610 (17.0)	365 (15.5)	1245 (17.5)	0.052
Risk scores				
HEAR	4 [2, 5]	4 [2, 5]	4 [3, 5]	0.180
HEART	4 [3, 6]	4 [2, 5]	4 [3, 6]	0.202
EDACS	14 [10, 18]	14 [10, 18]	14 [10, 18]	0.088
HEART components				
Item: History				0.276
Score 0	3674 (38.7)	1150 (48.9)	2524 (35.4)	
Score 1	1677 (17.7)	348 (14.8)	1329 (18.6)	
Score 2	4133 (43.6)	855 (36.3)	3278 (46.0)	
Item: EKG				0.063
Score 0	6045 (63.7)	1499 (63.7)	4546 (63.7)	
Score 1	1147 (12.1)	317 (13.5)	830 (11.6)	

Score 2	2292 (24.2)	537 (22.8)	1755 (24.6)	
Item: Age				0.060
Score 0	1326 (14.0)	318 (13.5)	1008 (14.1)	
Score 1	3587 (37.8)	942 (40.0)	2645 (37.1)	
Score 2	4571 (48.2)	1093 (46.5)	3478 (48.8)	
Item: Risk factors				0.098
Score 0	3720 (39.2)	976 (41.5)	2744 (38.5)	
Score 1	3944 (41.6)	990 (42.1)	2954 (41.4)	
Score 2	1820 (19.2)	387 (16.4)	1433 (20.1)	
Item: Initial troponin				0.174
Score 0	6009 (63.4)	1578 (67.1)	4431 (62.1)	
Score 1	2514 (26.5)	622 (26.4)	1892 (26.5)	
Score 2	961 (10.1)	153 (6.5)	808 (11.3)	
0-hour hs-cTnT, ng/L	11 [8, 18]	11 [8, 16]	11 [8, 18]	0.044
Index visit NSTEMI	657 (6.9)	75 (3.2)	582 (8.2)	0.216

Continuous variables were summarized as median (interquartile range) and categorical variable were summarized as number (percentage). SMD, standardized mean difference; BP, blood pressure; CAD, coronary heart disease; AMI, acute myocardial infarction; PCI, percutaneous coronary intervention; CABG, coronary artery bypass graft; EDACS, emergency department assessment of chest pain score; HEAR, history, electrocardiogram, age, and risk factors; HEART, history, electrocardiogram, age, risk factors, and troponin; ECG, electrocardiogram; hs-cTnT, high sensitivity-cardiac troponin T; NSTEMI, non-ST-elevation myocardial infarction.

Table S2. Calculation of HEART score.

Items	Score	Description
	2	Highly suspicious
**History**	1	Moderately suspicious
	0	Slightly or non-suspicious
	2	Significant ST-depression
**ECG**	1	Non-specific repolarization disturbance [1]
	0	Normal
	2	≥ 65 years
**Age**	1	45−65 years
	0	< 45 years
	2	≥ 3 or history of atherosclerotic disease [3]
**Risk factors** [2]	1	1 or 2 risk factors
	0	No risk factors known
	2	≥ 42 ng/L
**Hs-cTnT**	1	14-41 ng/L
	0	<14 ng/L

[1]Not ST deviation but left bundle branch block (LBBB), left ventricular hypertrophy (LVH), or other changes. [2]Risk factors includes hypertension, diabetes mellitus, hypercholesterolemia, smoking (current, or smoking cessation ≤3 months), and positive family history (parent or sibling with cardiovascular disease before age 65). [3]History of atherosclerotic disease includes prior myocardial infarction, coronary revascularization, cerebrovascular accident, and peripheral arterial disease. HEART, history, electrocardiogram, age, risk factors, and troponin; ECG, electrocardiogram; hs-cTnT, high sensitivity-cardiac troponin T.

Table S3. Calculation of emergency department assessment of chest pain score (EDACS).

Item and description	Score
**Male sex**	+6
**Age**	
18-45	+2
46-50	+4
51-55	+6
56-60	+8
61-65	+10
66-70	+12
71-75	+14
76-80	+16
81-86	+18
86+	+20
**Aged 18-50 years and either**	
known coronary artery disease [1] OR ≥ 3 risk factors [2]	+4
**Symptoms and signs**	
Diaphoresis	+3
Radiates to arm or shoulder	+5
Pain worsened with inspiration	-4
Pain is reproduced by palpation	-6

[1]Coronary artery disease includes previous acute myocardial infarction, coronary artery bypass graft, and percutaneous intervention. [2]Risk factors include dyslipidemia, diabetes, hypertension, current smoker, and family history of premature coronary artery disease.
